# *IGF2BP3* regulates *EMP1* stability in an m^6^A-dependent manner and activates the *TGF-β* pathway to promote pancreatic cancer invasion

**DOI:** 10.1038/s41419-025-08155-1

**Published:** 2025-11-24

**Authors:** Long Liu, Yu Zhang, Yuxi Huang, Xiaohong Zhao, Hongxing Fu, Lin Chen, Qi Wang, Xingyu Liu, Xiang Zheng, Hengqing Zhu, Zheping Fang, Zhenzhen Gao, Sheng Yan

**Affiliations:** 1 Department of Hepatobiliary and Pancreatic Surgery, The Second Affiliated Hospital of Zhejiang University School of Medicine, No. 88, Jiefang Road, Shangcheng District (Jiefang Road Hospital District), Hangzhou, Zhejiang Province China; 2 Department of Hepatobiliary and Pancreatic Surgery, Taizhou Hospital, Zhejiang University School of Medicine, China. No.150 Ximen Street, Linhai City, Taizhou City, Zhejiang Province China; 3 Department of Oncology, The First Affiliated Hospital of Anhui Medical University. 218 Jixi Road, Shushan District, Hefei City, Anhui Province China; 4 The First Hospital of the University of Science and Technology of China West District (Anhui Cancer Hospital). No. 107, East Huanhu Road, Shushan District, Hefei City, Anhui Province China; 5 Taizhou Hospital of Wenzhou Medical University. No.150 Ximen Street, Linhai City, Taizhou City, Zhejiang Province China; 6 School of Pharmacy, Hangzhou Normal University. No. 2318, Yuhangtang Road, Yuhang District, Hangzhou, Zhejiang Province China; 7 National University of Singapore School of Pharmacy. Block S4A, Level 3 18 Science Drive 4, Singapore, 117543 Singapore; 8 Department of Thyroid Surgery, Second Affiliated Hospital School of Medicine, Zhejiang University, Hangzhou, China

**Keywords:** Oncogenes, Cell invasion

## Abstract

Local invasion is considered a premonitor of tumor metastasis which cause curative difficulties and undesired prognosis in patients with Pancreatic ductal adenocarcinoma (PDAC). The importance of mRNA N6-methyladenosine (m^6^A) modification during tumor invasion is controversial as it plays distinct roles which mainly attributed to different m^6^A reader proteins exert function. In current study, the level of m^6^A expression in PDAC was analyzed by IHC and ELISA, all m^6^A-regulated genes in PDAC detected by qPCR. The downstream gene *EMP1* was screened by analyzing *IGF2BP3* knockdown RNA-seq, *IGF2BP3*-RIP, MeRIP-seq, and PDAC-survival related genes. And m^6^A modification sites of *EMP1* RNA was verified by MeRIP-qPCR, RIP-qPCR, and dual luciferase assays. *EMP1*-binding protein *VASP* was identified by mass spectrometry. Cell migration and invasive activity were detected using cytoskeletal staining, scratch assay, transwell assay, subcutaneous tumor and lung metastasis models. Finally, prognosis and immune microenvironment was analyzed in PDAC by IHC and multiple immunofluorescence staining. This work shows that m^6^A reader *IGF2BP3* is remarkably upregulated in local invasion PDAC and indicates worse prognosis of patients. Mechanistically, *IGF2BP3* recognized m^6^A-modified *EMP1* mRNAs to prolong stability of them, which inhibits the hindrance of *SMAD7* to *SMAD3/4* phosphorylation by promoting the binding of *VASP* and *SMAD7*. Finally, a tight correlation of the local invasion/*IGF2BP3/EMP1* and infiltration of immune cells in the tumor microenvironment is evidenced in clinical PDAC. In conclusions, *IGF2BP3* functions as an invasion driver that induces PDAC development via the *EMP1/TGF-β* axis. And *IGF2BP3/EMP1* axis may be involved in regulating microenvironmental remodeling in pancreatic cancer.

## Background

Pancreatic cancer is the third leading cause of cancer-related death in humans, and the mortality rate is still rising. It is projected to become the second leading cause of cancer-related mortality by 2030 [1, 2]. Currently, there is no validated screening method for early-stage tumors, and pancreatic cancer has a very poor prognosis. Therefore, most patients have a locally advanced state or metastasis at diagnosis. Surgery and cytotoxic therapy remain the mainstay of treatment for pancreatic cancer but have limited efficacy in patients with advanced pancreatic cancer [2]. Metastasis, specifically metastasis in the early stage, is a dominant cause of undesired prognosis and high mortality in patients with Pancreatic ductal adenocarcinoma (PDAC), and the main mechanisms of metastasis are categorized into local invasion and lymphatic metastasis [3]. This situation is induced by the complex crosstalk between tumor cells and microenviroment [4], which involves a multitude of factors such as angiogenesis/lymphangiogenesis, epithelial-mesenchymal transition (EMT), formation of pre-metastatic ecological niches, and dysregulation of the tumor immune microenvironment [3, 5, 6].

N6-methyladenosine, also known as m^6^A, was first discovered in 1974 and was found to be the most ubiquitous modification in the epitranscriptome of eukaryotic cells [7]. m^6^A is regulated by m^6^A methyltransferases, m^6^A binding proteins, and demethylases [8]. m^6^A and its related regulators play essential roles in cancer by affecting the stabilization, splicing, degradation, and translation efficiency of target genes [9, 10]. Therefore, m^6^A is a novel potential therapeutic target for pancreatic cancer. For example, the overexpression of *ALKBH5* can inhibit the proliferation and invasion of PDAC cells and improve the prognosis of patients with PDAC in combination with cytotoxic therapy since it can also increase the sensitivity of PDAC cells to gemcitabine treatment [11, 12]. However, the biological role of m^6^A in locally invasive pancreatic cancer has not been thoroughly investigated. *IGF2BP3*, as an m^6^A reader, is highly associated with the development, metastasis, and poor prognosis of many cancers, such as non-small cell lung cancer [13], colorectal cancer [14], and breast cancer [15]. The influence of *IGF2BP3* in the invasive capacity of pancreatic cancer cells still lacks in-depth studies.

The *EMP1* gene is located on the long arm of chromosome 12 (12p12.3) and encodes a hydrophobic tetra transmembrane transporter protein. According to previous reports, *EMP1* regulates cell proliferation and differentiation, and its expression of *EMP1* is specifically high in many cancers, such as gastric cancer [16], glioma [17], and colorectal cancer [18], indicating that *EMP1* is a novel therapeutic target for cancer therapy. In colorectal cancer, *EMP1* was expressed in a unique high-relapse tumor cell population, which is the main reason for metastatic recurrence of colorectal cancer after surgery [18]. Moreover, in glioblastoma, *EMP1* levels can be regulated by the epidermal growth factor receptor ligand, thereby contributing to the oncogenic role of the epidermal growth factor receptor in glioblastoma [19]. In glioblastoma, EMP1 has also been found to be a suppressor gene for killing function of T cells, which can be used to improve the effector function of T cells, increase the infiltration of T cells into the tumor site, and reduce the tumor growth by knocking down the *EMP1* gene in CD8 T cells [20]. However, the expression pattern of *EMP1* in PDAC and the role of the *IGF2BP3-EMP1* axis in the invasion of PDAC have not been revealed.

In this study, we observed that m^6^A levels were significantly elevated in invasive PDAC compared with samples without local invasion. Among the m^6^A-regulated molecules, *IGF2BP3* was the most significantly elevated and promoted pancreatic cancer cell invasion and proliferation by affecting the transforming growth factor-β (*TGF-β*) signaling pathway. Furthermore, bioinformatics analysis and experimental validation revealed that *EMP1* mRNA contains functional m^6^A methylation sites. The m^6^A reader *IGF2BP3* binds to these sites and stabilizes *EMP1* mRNA, enhancing its expression in pancreatic cancer. Moreover, we analyzed the role of the *IGF2BP3-EMP1* axis in the regulation of the tumor immune microenvironment by multiple immunofluorescence of PDAC. Our study expands the understanding of m^6^A methylation modifications in PDAC invasion and immune regulation, providing a promising new molecular basis for the development of inhibitors that suppress the early invasion and metastasis of PDAC.

## Materials and methods

### Cell culture

Pancreatic cancer cell lines, AsPC-1 and PANC-1, were obtained from the Innovation Center of Excellence in Molecular Cell Science, Chinese Academy of Sciences (Shanghai, China), incubated in Dulbecco’s Modified Eagle Medium (DMEM, Gibco, New York, USA) supplemented with 10% fetal bovine serum (CellMax, China) and 1% penicillin-streptomycin in a humidified 5% CO_2_ incubator at 37 °C, and tested negative for mycoplasma contamination.

### Clinical specimens

Cohort1 consists of 80 pairs of pancreatic cancer and paracarcinoma paraffin tissues including information on patients’ follow-up overall survival time. Cohort2 consisted of 331 patients with pancreatic cancer and was used to analyze the relationship between local invasion of pancreatic cancer and recurrence-free survival. Eight fresh pancreatic cancer samples were used for single-cell sequencing, and eight pairs of frozen pancreatic cancer with or without local invasion were used for PCR and Western blot analysis. Cohort1 consists of 80 pairs of pancreatic cancer, collected from our center, were also constructed tissue microarrays for analysis of *METTL3, IGF2BP3, EMP1* and microenvironmental markers. In addition, we downloaded the TCGA-PAAD dataset (*N* = 183) from TCGA (The Cancer Genome Atlas) for *METTL3, IGF2BP3* and *EMP1* survival and correlation analysis. All pancreatic cancer and paracancerous samples were obtained from the Second Affiliated Hospital of Zhejiang University School of Medicine. With the written informed consent from the patient, the Second Affiliated Hospital of Zhejiang University School of Medicine and the Ethics Committee of the hospital approved (number: 2024-0547) the collection of specimens and patient information, including age, gender, TNM stage (Including status of tumor size, lymph node metastasis and distant metastasis), and survival.

### RNA extraction, RT-qPCR, and RNA stability

Total RNA was extracted using RNAiso Plus (TaKaRa, China), followed by cDNA synthesis using a PrimeScriptTM RT Reagent Kit (TaKaRa, China), following the manufacturer’s instructions. Real-time PCR (RT-PCR) was conducted using TB Green® Premix Ex TaqTM II (TaKaRa, China) by an ABI Prism 7500 System (Applied Biosystems, Carlsbad, CA, USA). Relative gene expression was calculated by the 2^-ΔΔCT^ method with *GAPDH* as the internal control. Primer Sequences are provided in Supplementary Table S1.

### Western blot analysis

Cell and tissue specimens were rinsed twice using ice-cold phosphate-buffered saline (PBS), and the total proteins were extracted with RIPA buffer (Beyotime, P0013B, China) that consisted of 5 mM EDTA, PMSF, a cocktail phosphatase inhibitor, followed by protein concentrations determination through a BCA kit (Beyotime, China). Then, the total cell lysates were separated by SDS-PAGE and transferred to PVDF membranes, followed by blocking with 5% skim milk. Membranes were then incubated with *IGF2BP3* (Abcam, ab179807, US), *GAPDH* (Proteintech, 60004-1-Ig, China), *EMP1* (Abcam, ab230445, US), *VASP* (Proteintech,13472-1-AP, China), *SMAD2* (Proteintech,12570-1-AP, China), *SMAD3* (Proteintech,66516-1-1 g, China), *SMAD7* (Proteintech,25840-1-AP, China), *phospho-SMAD2* (CST,138D4, US), and *phospho-SMAD3* (CST, 9520S, US) at 4 °C overnight. Then, the signal was visualized through the ECL chromogenic kit (Beyotime, P0018S, China) on the Mini-REPORT Tetra Electrophoresis System(all uncropped membranes in Fig. S3). The intensity of each band was quantified using ImageJ software (National Institutes of Health). *GAPDH* was used as the internal control.

### Mass spectrometry analysis

Pancreatic cancer cells were cultured and lysed for total protein extraction. Proteins were precipitated by agarose beads with IgG antibody or anti-EMP1 antibody and silver-stained on SDS-PAGE gels. Total proteins that were not immunoprecipitated were used as the “input” group (positive control). After electrophoresis, the regions with the most significant differences between the IgG and EMP1 groups were excised and analyzed by mass spectrometry.

### Immunohistochemistry assay

Paraffin-embedded tissue sections were deparaffinized, rehydrated, and boiled in 10 mm citrate buffer (pH = 6.0) for 30 min for antigen extraction. Subsequently, 3% hydrogen peroxide was added and incubated for 15 min to eliminate endogenous peroxidase. The tissues were cultured in goat serum at room temperature. After incubation for 30 min, tissues were cultured with anti-m^6^A (Proteintech, 68055-1-Ig, China), anti-*IGF2BP3* (ab179807#5246, Abcam, USA), and anti-*EMP1* (Abcam, ab230445, USA) antibodies at 4 °C overnight. Immunodetection was performed the next day using DAB (Servicebio, G1212-200T, China) according to the manufacturer’s instructions. Two independent observers determined the staining scores using the ImageJ software based on the proportion of indicated protein-positive cells and brown color intensity.

### ScRNA-seq downscaling and unsupervised clustering

Single cell RNA-seq library constructs were prepared using 10× Genomics Chromium Single Cell 3ʹ Reagent Kits v3 according to the manufacturer’s instructions. After QC and filtering, the feature barcode matrices of each library were processed by Seurat (version 4.0.4) [21]. The R package was used for scaling and linear dimensionality reduction. For visualization, the dimensionality of each dataset was further reduced using the Uniform Streamform Approximation and Projection (UMAP) of the Seurat function RunUMAP [22]. For unsupervised clustering, k-nearest neighbors based on Euclidean distance in PCA space are first computed and shared nearest neighbor graphs are constructed from find neighbors. Then, the modular optimization technique of the Leuven algorithm is applied to identify cell clusters [23].

### Stable cell construction

Lentiviruses, which were designed together with shRNAs against *IGF2BP3*, *EMP1*, or negative control RNAs (shCtrl), and lentiviruses expressing *EMP-1-*flag, *IGF2BP3*-flag, empty vector, were constructed by Genechem (Shanghai, China). The small interfering RNA for *METTL3* was constructed by Ribio (Guangdong, China). Briefly, 1.5 × 10^5^ cells were cultured in a six-well plate; then, the lentiviruses were seeded for transfection according to the instructions (MOI = 30). After that, the incubator was filtrated for one week with 3–5 µg/mL puromycin (MCE, HY-15695, USA), followed by a determination of the efficiency of transfected cells.

### Wound-healing assay

PANC-1 and AsPC-1 cells transfected with siRNA for 24 h or lentivirally transduced stable cells were incubated in 6-well plates until they reached confluency. The cells were then scraped using a P200 pipette tip (0 h), rinsed with PBS, and incubated in serum-free DMEM. After 24 h, three images with non-overlapping fields of view were obtained. Cell migration rate = (initial scratch width - final scratch width)/initial scratch width × 100%, the wound area was quantified using ImageJ software.

### Phalloidin staining F-actin assay

Transfected PANC-1 and AsPC-1 cells were cultured in 48-well plates for 24 h per well with 300 µL Alexa Fluor 555 labeled phalloidin (Beyotime, C2203S, China). After fixing and washing with PBS, nuclei were stained with DAPI for 5 min and observed under a fluorescence microscope.

### Cell invasion and migration assay

To analyze cell invasion and migration, cells were seeded in the upper chamber of Transwell plates (Corning Incorporated, New York, USA) coated or uncoated with Matrigel (BD Biosciences, New Jersey, USA) and cultured for 48 h. In the invasion experiments, serum-free medium was used in the upper chambers and medium containing 10% fetal bovine serum (FBS) was used in the lower chambers. The total cells were fixed in 4% paraformaldehyde for 20 min and visualized by staining with 0.1% crystal violet for 20 min. Migrating and invading cells were observed under a microscope, and the cells were quantified in three fields of view using the ImageJ software.

### m^6^A enzyme-linked immunosorbent assay (ELISA)

The amount of m^6^A was detected using a specific capture m^6^A antibody and detection antibody in an RNA m^6^A quantification kit (ab185912, Abcam, USA). First, total RNA was added to the incubator along with the capture antibody and cultured for 1 h. The detection antibody and enhancer solution were then added to enhance the detected signal. After incubation with the developer solution, the detected signal was quantified by measuring the absorbance in a microplate spectrophotometer at a wavelength of 450 nm.

### RNA immunoprecipitation assay

An RNA-binding protein immunoprecipitation kit (17-700, Millipore, USA) was used to perform the RIP assay, following the manufacturer’s instructions. First, cells were collected and resuspended in RIP lysate, and then the cell lysate was dispensed in an Eppendorf tube. The cell lysate was then added to the tube, together with the magnetic beads and 5 μg of mouse immunoglobulin G (17-700, Millipore, USA) or Flag (Proteintech, 66008-4-Ig, China), and cultured for 3 h. Protein-RNA complexes were then extracted using a magnetic separator, and the supernatant was discarded. The immunoprecipitate was incubated with proteinase K buffer, followed by RNA purification. Finally, the RNA sequence that interacted with the *IGF2BP3-flag* was identified by qPCR.

### Methylated RNA immunoprecipitation-qPCR

Total RNA was extracted and purified using an mRNA purification kit (61,006, Invitrogen, USA), fragmented using a sonicator, and 2 μg of the RNA was saved as the input at −80 °C. Next, the remaining RNA was added to the tube, together with an anti-m^6^A antibody (68055-1-Ig, Proteintech, China), and immunoprecipitation was performed. The A/G magnetic beads (88,803, Thermo, USA) were added for 1 h incubation. Finally, the eluted RNA and input were reverse transcribed, and qPCR was performed using the designed specific m^6^A primers.

### Dual-luciferase reporter assay

To perform the m^6^A dual luciferase reporter experiment, sh-*IGF2BP3*, sh-Control, vector, and specific overexpressing *IGF2BP3* PDAC cells were first inoculated in triplicate the day before transfection. To analyze the *EMP1* promoter dual-luciferase reporter, the wild-type *EMP1* promoter region or mutant1 or mut2 of the *EMP1* 3’UTR promoter region was plugged into the reporter plasmid pGL3-Basic, 50 ng of wild-type or mutant firefly luciferase reporter plasmid along with 5 ng of Renilla luciferase plasmid (Promega, Madison, WI, USA). Then, total cells were co-transfected with the reporter plasmid carrying the wild-type, mut1 or mut2 of *EMP1* 3’UTR, together with the Renilla luciferase (Rluc) control plasmid (pRL-TK), using Lipofectamine 2000 (11668030, Invitrogen, USA). After transfection, total cells were cultivated for 48 h, followed by firefly luciferase (Fluc) and Rluc activities calculations using the Dual-Luciferase Reporter Analysis System (Promega). The fluorescence value was read on a fluorescence microplate reader immediately after mixing. Then the Renilla luciferase working solution was added, and then read the fluorescence value. The Renilla fluorescence was used as an internal reference to calculate the firefly fluorescence change in each group.

### Prediction of potential binding sites for VASP-SMAD7

SMAD7 (ID: O15105), VASP (PDB ID: 1EGX) protein structures were obtained from UniProt database (https://www.uniprot.org/) and RCSB database (https://www.rcsb.org/). All protein structures to were processed in the Molecular Operating Environment (MOE 2019.1) platform with the stance choice of Amber10, including removal of water and ions, protonation, addition of missing atoms and complementation of missing groups, and protein energy minimization.The HDOCK server predicts binding complexes between two molecules, such as proteins and nucleic acids, through the use of hybrid docking strategies. Using the HDOCK software, each protein was set to be rigid, the docking contact site was set to be full-surface, the resulting conformation after docking was set to be 100, and the docking score was calculated based on the knowledge-based iterative scoring function ITScorePP. The optimization results were most subsequently visualized using Pymol 2.1 software to graphically analyze the model.

### Animal experiments

BALB/c nude mice (six-week-old) were bought from the Laboratory Animal Center of the Chinese Academy of Sciences (Shanghai, China). Animal experiments was approved by the Second Affiliated Hospital of Zhejiang University School of animal Ethics Committee of the hospital approved (number: 2024-057) and randomly divided into several groups. Next, 5 × 10^6^ stably transfected PDAC cells were resuspended in 100 μL PBS and subcutaneously injected into the left abdomen of mice. The tumor size was measured at intervals of several days until six weeks or more. After that, the mice were euthanized, Tumor weights were then measured for histological analysis. Finally, the tumor growth curve was estimated based on tumor volume calculated by the “length × width 2/2” formula. For the lung metastasis model, lentivirus packaged from an empty lentiviral vector or control shRNA plasmid was used to infect PANC-1 cells. Nude mice were injected with these cells in the tail vein at a dose of 5 × 10^6^ cells/mouse. Six weeks after tumor cell injection, BALB/c nude mice were euthanized to obtain subcutaneous tumors or lung tissues for evaluation.

### Data availability and analysis

Then, htseq (version 0.11.2) was used to generate raw counts, and HISAT2 (version 2.1.0) was used to map the reads to the human genome (version hg19). After that, the differentially expressed genes were calculated by the R package DESeq2 (version 1.28.1). Followed by producing the volcano plots of these differential expression genes using the R package ggplot2 (version 3.3.0). For gene set enrichment analysis, normalized enrichment scores were calculated, and nominal *p*-values were compared. Bioinformatic analysis of visual heatmaps was performed using the MeV program (version 4.4). The total datasets were obtained from TCGA-PAAD and NCBI GEO (http://www.ncbi.nlm.nih.gov/geo/) under the accession numbers: GSE205013, GSE190078 and GSE79147.The data that support the findings of this study are available from the corresponding author upon reasonable request.

### Statistical analysis

Statistical analysis was conducted using Statistical Package for the Social Sciences software (version 22.0, Chicago, IL, USA). Unless otherwise stated, data are reported as either mean ± standard deviation of the mean of at least three independent experiments. All data were first assessed for normality using the Shapiro-Wilk test. For normally distributed data, a two-tailed Student’s t-test (for two groups) or one-way analysis of variance (for multiple groups) was used. For comparisons where variance homogeneity was not met (assessed by Levene’s test), Welch’s t-test was used instead. When normality assumptions were violated, the nonparametric Mann-Whitney U test was employed. The survival curveswere plotted using the Kaplan-Meier method. The *P* value < 0.05 was considered statistically significant.

## Results

### *IGF2BP3* activated the *TGF-β/Smads* signaling pathway to promote PDAC invasion and poor prognosis

**Fig. 1:**
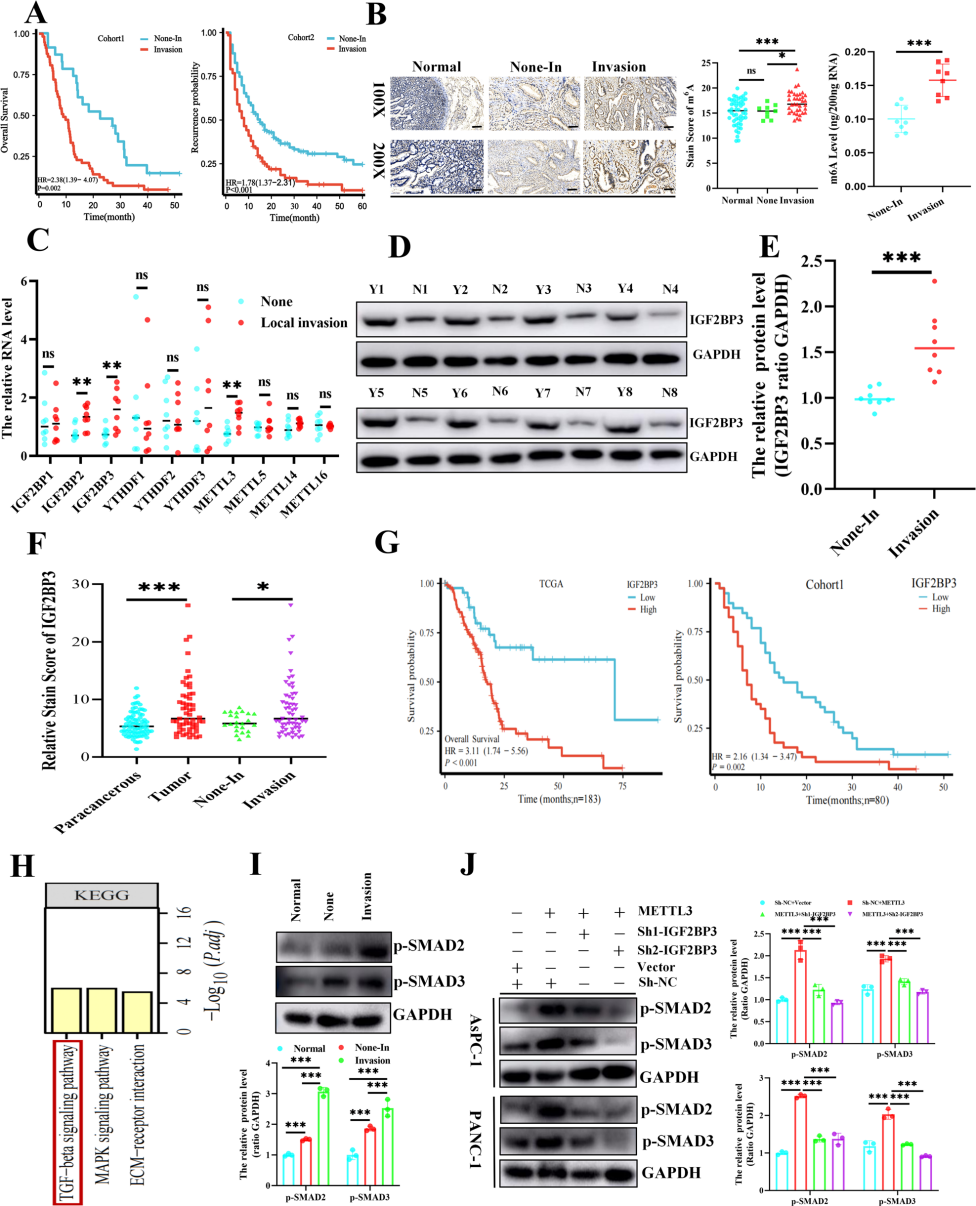
*IGF2BP3* activates the *TGF-β/Smads* signaling pathway to promote pancreatic cancer invasion and poor prognosis dependent on m^6^A modifications. Local invasion is associated with poor prognosis in pancreatic cancer (**A**); Levels of m^6^A in locally invasive and non-invasive pancreatic cancer tissues were analyzed by IHC and ELISA (**B**)**;** Levels of main m^6^A-related genes in locally invasive pancreatic cancer and non-invasive pancreatic tissues was detected by qPCR (**C**)**;** Protein levels of *IGF2BP3* in nonlocally invasive (“N”), and locally invasive (“Y”) pancreatic cancer tissues, was detected by Western blot (**D**), (**E**) and IHC (**F**)**;** Impact of *IGF2BP3* expression levels on overall survival in pancreatic cancer patients was analyzed in *TCGA-PAAD* and our center (**G**)**;** KEGG functional enrichment analysis of the Sh-NC versus Sh-*IGF2BP3* PANC-1 dataset (**H**)**;** Western blot analysis of the main markers associated with *TGF-β/Smads* activation in human pancreatic cancer tissues (**I**) and pancreatic cancer cells (PANC-1 and AsPC-1) (**J**). Scale: 100 μm. *, *P* < 0.05; ***, *P* < 0.001.

m^6^A plays an essential role in tumor invasion and metastasis [24]. Advanced invasive and metastatic abilities are typical features of pancreatic adenocarcinoma [4]. Among them, local invasion is considered an important reason why pancreatic cancer is surgically lost at the early stage of first detection and is also associated with recurrence and poor overall survival time (Fig. 1A). We evaluated the expression levels of m^6^A in pancreatic cancer tissues that underwent local invasion or none using Immunohistochemistry (IHC) and Enzyme Linked Immunosorbent Assay (ELISA) (Fig. 1B) and observed that the expression levels of m^6^A were elevated in local invasive pancreatic cancer tissues. Subsequently, we analyzed the expression of m^6^A-related genes in local invasive or non-invasive pancreatic cancer tissues by qPCR. We found that among the numerous m^6^A-associated genes, *IGF2BP3*, an m^6^A-reading protein, had the most significant increase (Fig. 1C), which was consistent with the protein levels in pancreatic cancer (Fig. 1D, E). The protein level of *IGF2BP3* was evaluated by IHC in pancreatic cancer tissue microarrays (Cohort1). We found that the *IGF2BP3* expression was significantly elevated in pancreatic cancer compared with non-cancerous tissue. Moreover, *IGF2BP3* expression was significantly higher in pancreatic cancer tissues with localized tissue invasion than samples without localized invasion (Fig. 1F). Besides these, high expression of *IGF2BP3* predicted poorer overall survival time by analyzing pancreatic cancer samples from *TCGA-PAAD* and our center (Fig. 1G). Besides, we also analyzed the level of *METTL3* protein in locally invasive pancreatic cancer tissues compared to controls using Western blot, the results showed that *METTL3* protein was significantly highly expressed in pancreatic cancer tissues with localized tissue invasion. In addition, statistical analysis of pancreatic cancer microarrays showed that *METTL3* was associated with poorer overall survival time (Fig. S1A, B). Subsequently, the Kyoto encyclopedia of genes and genomes (KEGG) functional enrichment analysis differential genes (Fig. S1C) of RNA-seq data from pancreatic cancer cells with *IGF2BP3* knockdown revealed that the *TGF-β/Smads* signaling pathway was predominantly affected (Fig. 1H). A subsequent assay of proteins from pancreatic cancer tissue samples (Fig. 1I) and cell samples (Fig. 1J) once again corroborated the effect of *IGF2BP3* on *TGF-β/Smads* pathway activation.

### *IGF2BP3* promoted the metastatic and invasive ability of pancreatic cancer cells in vivo and in vitro experiments

**Fig. 2:**
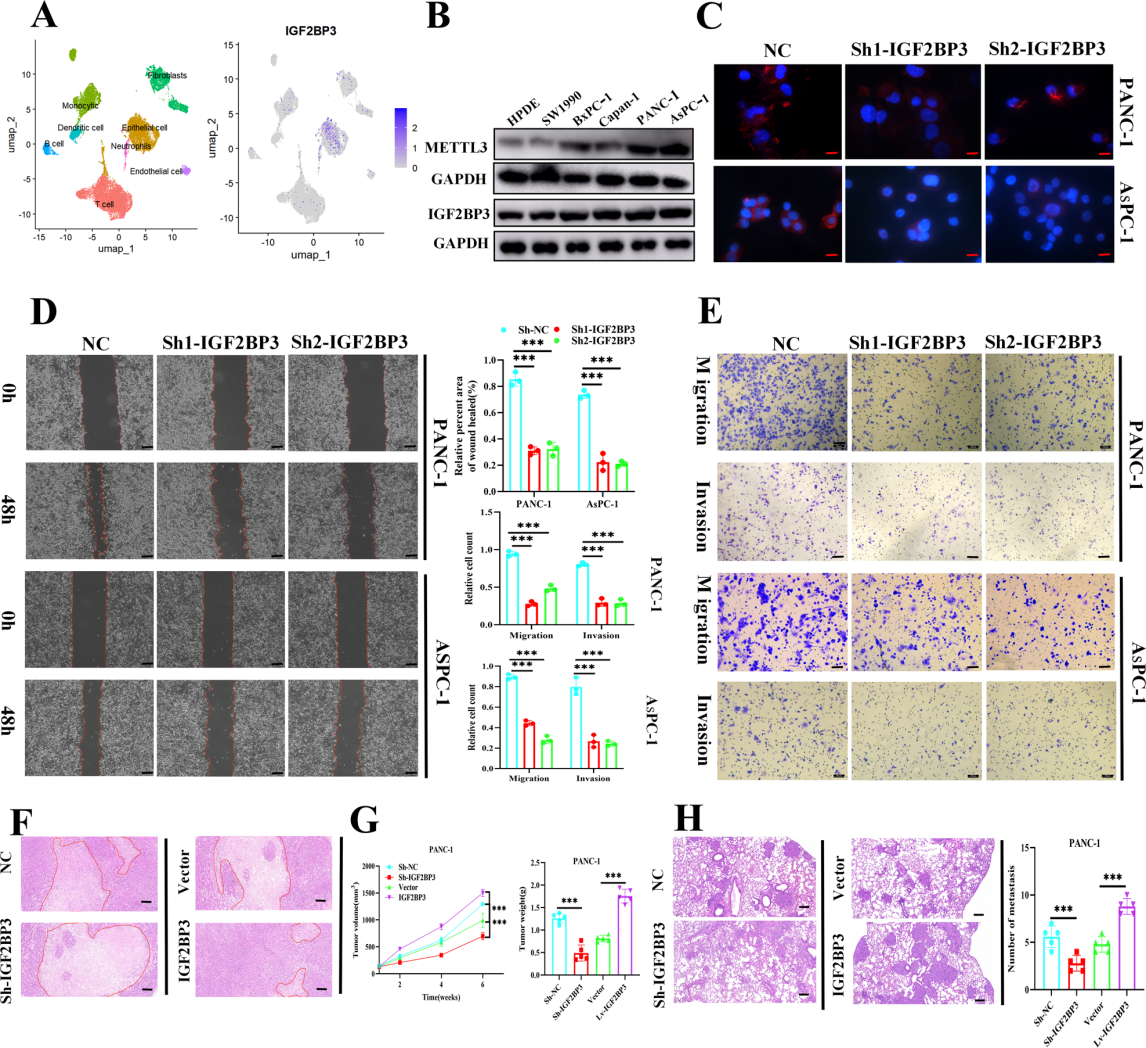
*IGF2BP3* promotes metastatic and invasive ability of pancreatic cancer cells in vivo and in vitro. Pancreatic cancer single-cell sequencing data reveal subcellular expression of *IGF2BP3* in pancreatic cancer (**A**)**;** Western blot was used to detect the expression level of *IGF2BP3* and *METTL3* in human pancreatic cancer cell line (**B**) Sh-control, *Sh1-IGF2BP3*, and *Sh2-IGF2BP3* pancreatic cancer cell lines (PANC-1 and AsPC-1) were constructed to assess the invasive ability of pancreatic cancer cells: cytoskeleton staining (**C**) scratch assay (**D**) Transwell migration and invasion assay (**E**)**;** Hematoxylin and eosin (H&E) staining depicting morphology (**F**) and tumor proliferation curves (**G**) and tumor weight (**G**) of subcutaneous tumor xenografts formed by the indicated cells, (1 × 10^6^), *n* = 5; H&E staining (**H**) lung metastases formed by injection of the indicated cells through the tail vein, and provides the number of lung surface nodules and metastatic lesions in mouse lung tissue (**H**). *n* = 5. Scale bar: 100 μm. ***, *P* < 0.001.

The tumor is a complex composed of tumor cells, immune cells, stromal cells, and extracellular matrix, and genes expressed in different cells may play different functions [25]. For this reason, We collected human pancreatic cancer tissue samples for single-cell sequencing, and annotated the results with degradation and dimension (Fig. S1D). We obtained a total of 8 cell populations including: T cells, B cells, monocytes, neutrophils, fibroblasts, endothelial cells, and epithelial cells. Subsequently, we mapped the *IGF2BP3* expression in various cellular subpopulations found that *IGF2BP3* was expressed predominantly in tumor cells compared to other cell groups (Fig. 2A). Subsequently, we evaluated the expression levels of *IGF2BP3, METTL3 and m*^*6*^*A* levels in several pancreatic cancer cell lines using Western blot and ELISA, The results showed that *METTL3* and *IGF2BP3* were mainly expressed in PANC-1 and AsPC-1 cells, compared to SW1990, BxPC-1, and Capan-1, and found a significant correlation between both *METTL3* protein levels and m^6^A-modified mRNA levels and *IGF2BP3* protein in pancreatic cancer cells (Fig. 2B, S1E). To further explore the effect of *IGF2BP3* on the invasive and metastatic abilities of pancreatic cancer cells, we constructed control, *Sh-IGF2BP3* (including two targets: Sh1 and Sh2), vector, and *IGF2BP3* pancreatic cancer cell lines for a series of in vivo and in vitro experiments. We identified that when *IGF2BP3* expression was suppressed, the skeleton synthesis in pancreatic cancer cells was inhibited (Fig. 2C). The metastatic and invasive abilities of pancreatic cancer cells were impaired in scratch (Fig. 2D) and Transwell migration and invasion assays (Fig. 2E). Finally, we established a subcutaneous xenograft tumor and a tail vein injection lung metastasis model in nude mice. We observed that *IGF2BP3* promoted proliferation in vivo (Fig. 2F, G; Fig. S1F) and metastatic invasive ability (Fig. 2H; Fig. S1F) in pancreatic cancer. Moreover, to explore the effects of the *METTL3-IGF2BP3* axis on the metastatic and invasive ability of pancreatic cancer cells, we performed a number of series of in vivo and in vitro experiments. The results showed that knockdown of METTL3 inhibited the migration and invasive ability of pancreatic cancer cells, and overexpression of *METTL3* would have an effect on the promotion of pancreatic cancer cell migration and invasive ability. In addition, knockdown of *METTL3* eliminated the effect of IGF2BP3 overexpression, and consistently, *IGF2BP3* silencing eliminated the effect of *METTL3* overexpression (Fig. S1G, H). These findings reveal the important role of *METTL3*-*I*GF2BP3 axis in the invasive and metastatic ability of tumor cells in pancreatic cancer and provide a basis for the clinical targeting of *IGF2BP3* to inhibit the invasive and metastatic ability of pancreatic cancer.

### *IGF2BP3* activated the *TGF-β/Smads* signaling pathway in an m^6^A-dependent manner by promoting the stability of *EMP1* mRNA levels

**Fig. 3:**
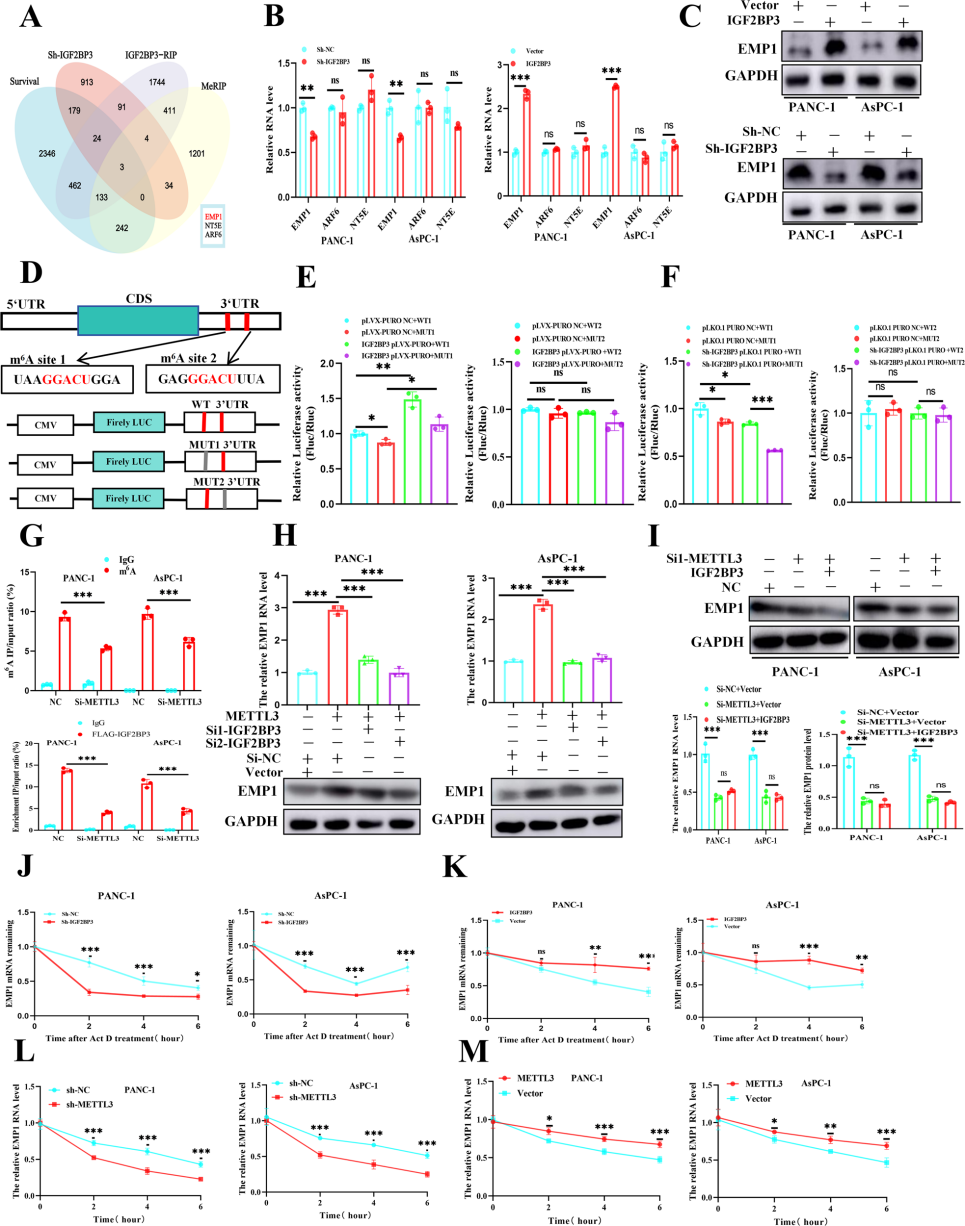
*IGF2BP3* activates the *TGF-β/Smads* signaling pathway in an m^6^A-dependent manner by promoting the stability of *EMP1* mRNA levels. Venn diagram demonstrating overlapping of MeRIP-seq dataset of *METTL3*-silenced cells, *IGF2BP3*-RIP-seq, Sh-*IGF2BP3*-RNA-seq dataset, and survival associated gene in pancreatic cancer (**A**); qPCR (**B**) and Western blot (**C**) detection of three overlapping genes in PANC-1 and AsPC-1 in the presence of *IGF2BP3* silencing; Schematic representation of the fusion of wild-type or mutant (mut1 and mut2) *EMP1* with the fluc reporter gene, luciferase activity was affected by wild-type or mutant (mut1 and mut2) *EMP1* (**D–F**); MeRIP-qPCR assay revealed enrichment of m^6^A modifications in the *EMP1* 3’UTR in control (NC) or *METTL3*-silenced PANC-1 and AsPC-1 cells (**G**)**;** RIP-qPCR assay for the binding of *IGF2BP3* to the *EMP1* mRNA 3 ‘UTR (**G**)**;** *EMP1* levels were detected by qRT-PCR and Western blot method under the intervention of *IGF2BP3* and *METTL3* (**H**, **I**); The qRT-PCR assay revealed the stability of *EMP1* mRNA under *METTL3*-*IGF2BP3* knockdown or overexpression (**J-M**).*, *P* < 0.05; **, *P* < 0.01; ***, *P* < 0.001.

To further elucidate the molecular mechanisms by which *IGF2BP3* promotes metastasis and invasion in pancreatic cancer, we comprehensively analyzed the MeRIP-seq dataset of pancreatic cancer cells with *METTL3* silencing, the *IGF2BP3*-RIP-seq, Sh-*IGF2BP3*-RNA-seq datasets, and pancreatic cancer prognosis-related differential gene clusters (Fig. 3A), *EMP1*, *NT5E*, and *ARF6* were obtained as target genes downstream of *IGF2BP3*. However, in further *IGF2BP3* knockdown experiments in PANC-1 and AsPC-1 cells, it was revealed that only the level of *EMP1* decreased consistently with *IGF2BP3* knockdown, and *IGF2BP3* overexpression received consistent results (Fig. 3B). The same result was corroborated at the protein level of *EMP1* (Fig. 3C, S1K, L). These results prompted us to choose *EMP1* as the downstream target gene of *IGF2BP3* for further studies. Considering that the important biological function of *IGF2BP3* is to play as a “reader” for m^6^A to promote gene stability and expression, we speculated that the regulation of *EMP1* by *IGF2BP3* might be m^6^A-dependent manner. The possible m^6^A modification sites of *EMP1* were predicted using SRAMP (an online m^6^A site predictor) [26]. The results of the previous analyses were consistent with the fact that the “very high” site of m^6^A modification of *EMP1* was in the 3’UTR region, and the two “very high” sites of *EMP1* in the 3’UTR were selected for further study (Fig. 3D, S1I). First, dual-luciferase reporter gene experiments revealed that *IGF2BP3* expression significantly increased the activity of *EMP1* mRNA containing the wild-type m^6^A modification, but not the mutant, and that the association of *IGF2BP3* with the mRNA region of the *EMP1* m^6^A modification could be eliminated by mutations transfected into its hypothesized m^6^A modification sites (mut1) (Fig. 3D-F). In addition, for further assessment to evaluate the binding of *IGF2BP3* to *EMP1* mRNAs with or without mutations (mut 1 and mut 2) at the m^6^A modification site, we synthesized Biotin-*EMP1*-mut1, Biotin-*EMP1*-mut2, Biotin-ss-RNA(negative control) and Biotin-ss-m^6^A RNA(positive control) sequences, and detected the binding between the *IGF2BP3* protein and them by RNA pull-down, and the results showed that the *IGF2BP3* protein could bind to Biotin-EMP1-mut2, Biotin-ss-m^6^A but not, Biotin-EMP1-mut1, which again demonstrated the presence of m^6^A modification in EMP1 site1(Figure S1J). And then we then constructed primers specific for detecting the selected “very high” m^6^A modification sites of *EMP1*. The m^6^A modifications at the selected “very high” site were confirmed by MeRIP-qPCR in the 3’UTR region of *EMP1* mRNA (Fig. 3G). Moreover, the recognition and binding of *IGF2BP3* to the m^6^A site of *EMP1* were detected by *IGF2BP3*-RIP-qPCR (Fig. 3G). *METTL3* regulated m^6^A modification, and *IGF2BP3* recognized the 3’UTR region of *EMP1* mRNA, which could be eliminated by silencing *METTL3* in pancreatic cancer cells (Fig. 3G). We further demonstrated that silencing of *IGF2BP3* reversed *METTL3*-induced upregulation of *EMP1* in pancreatic cancer cells, and we also examined the mRNA and protein levels of EMP1 after knockdown of METTL3 in pancreatic cancer cells and found that it was not reversible after overexpression of *IGF2BP3*, demonstrating the role of the *METTL3-IGF2BP3* axis in the regulation of *EMP1* at the RNA and protein levels, as detected by qPCR and Western blotting (Fig. 3H, I, S1M). Additionally, *IGF2BP3* overexpression was significantly prolonged, whereas silencing *IGF2BP3* shortened the half-life of *EMP1* mRNA (Fig. 3J, K). Furthermore, we analyzed the effects on *EMP1* mRNA stability after *METTL3* knockdown or overexpression and showed that overexpression of *METTL3* in pancreatic cancer cells increased the stability of *EMP1* mRNA, and consistently, the stability of *EMP1* mRNA was inhibited after *METTL3* knockdown (Fig. 3L, M). Taken together, our results demonstrate the important role of *METTL3-IGF2BP3*, an m^6^A modification-recognition regulation combination, in activating the *TGF-β/Smads* signaling pathway by promoting the stability of *EMP1* mRNA and upregulating the expression of *EMP1* in pancreatic cancer.

### Elevated *EMP1* was associated with poor prognosis in PDAC

**Fig. 4:**
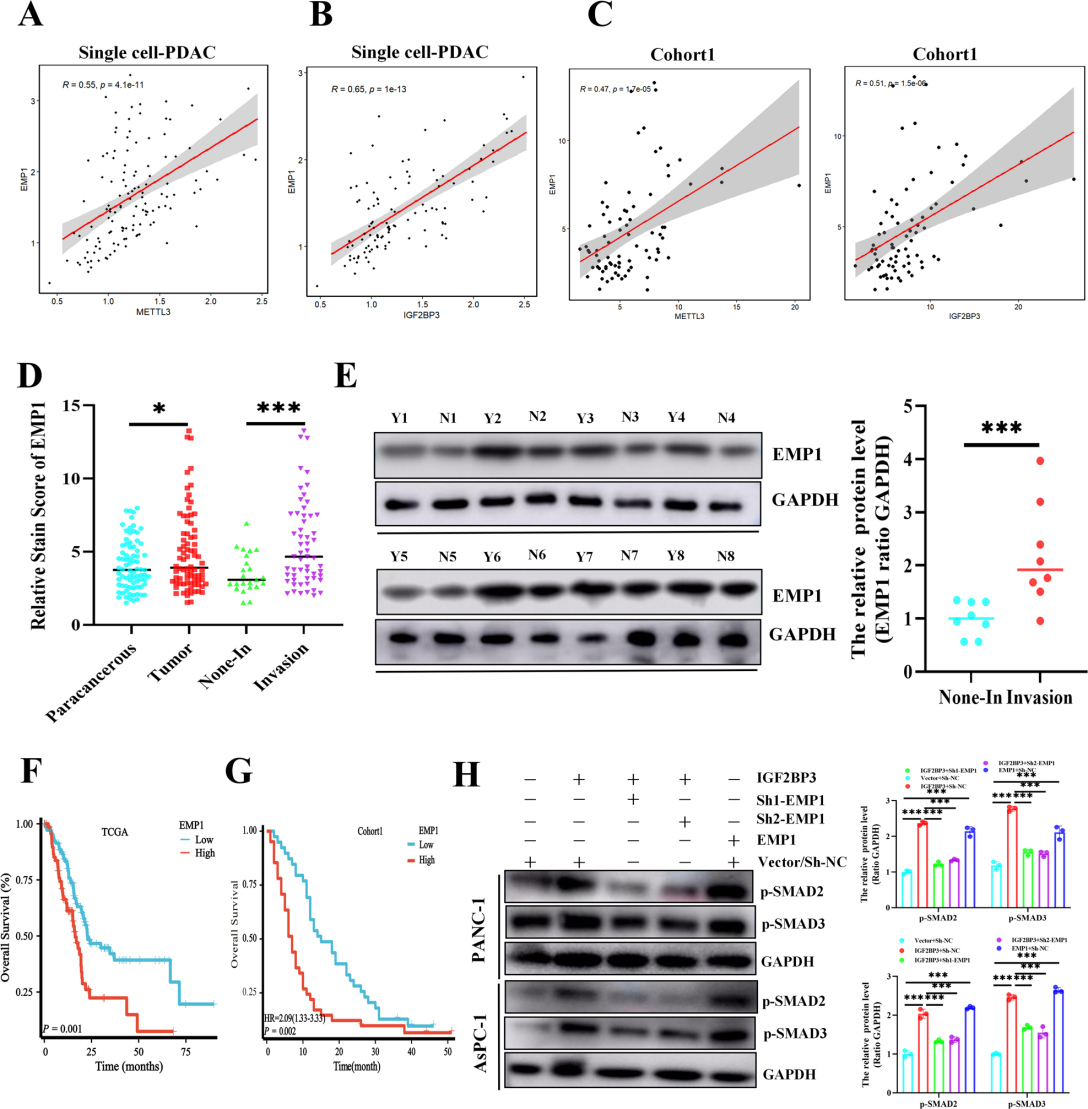
Elevated *EMP1* was associated with poor prognosis in PDAC. Correlation between *IGF2BP3, METTL3*, and *EMP1* expression in single-cell sequencing datasets (**A**, **B**) and Cohort1 (**C**), and Both *METTL3* and *IGF2BP3* were significantly positively correlated with *EMP1*. Expression of *EMP1* in non-invasive or locally invasive pancreatic cancer and matched paracancerous tissues (Cohort1, *n* = 80) was detected by IHC (**D**). Western blot analysis of *EMP1* protein in PDAC primary tumors from eight non-invasive or locally invasive samples (**E**); *EMP1* levels were analyzed for overall survival in pancreatic cancer samples (**F**, **G**); Western blot detected the influence of *EMP1* on *TGF-β pathway* (**H**). *, *P* < 0.05; ***, *P* < 0.001.

First, we quantified the expression of *EMP1*, *IGF2BP3* and *METTL3* in cell subpopulations of pancreatic cancer by analyzing single-cell sequencing data (Fig. S2A). Subsequently, we analyzed the correlation among *METTL3* and *IGF2BP3* and *EMP1* in the pancreatic cancer single-cell dataset. First, we filtered out tumor cells expressing only one gene or none, using cells expressing both genes to assess correlation. The results revealed a significant positive correlation between the expression of *METTL3* and *IGF2BP3* and *EMP1* in tumor cells (Fig. 4A). Besides, we detected the expression of *METTL3* and *EMP1* in pancreatic cancer samples serially sectioned with IGF2BP3 in Cohort1 and found that *EMP1* was also positively correlated with *METTL3* and IGF2BP3 (Fig. 4C). Moreover, in pancreatic cancer, *EMP1* protein levels were significantly higher in pancreatic cancers than in paracancerous tissues, as well as in pancreatic cancer samples with local invasion compared to those without local invasion (Fig. 4D). Subsequent Western blotting consistently demonstrated that *EMP1* was highly expressed in pancreatic cancer tissues that underwent localized invasion (Fig. 4E). High expression of *EMP1* was associated with poorer overall survival, both in *TCGA-PAAD* and in pancreatic cancer tissue microarrays (Fig. 4F, G). Finally, we analyzed the modulation of the *TGF-β* signaling pathway by *IGF2BP3*-*EMP1*. And found *EMP1* overexpression activates the TGF-β signaling pathway (Fig. 4H). *IGF2BP3* overexpression in pancreatic cancer cells promoted the activation of the *TGF-β* signaling pathway. However, this was reversed after knocking down *EMP1* (Fig. 4H). Together, these results reveal that elevated *EMP1* is associated with greater invasiveness and poorer survival and that *EMP1* and *IGF2BP3* expression are positively correlated.

### *IGF2BP3* combined with *EMP1* regulated the metastatic and invasive abilities of pancreatic cancer cells in vivo and in vitro

**Fig. 5:**
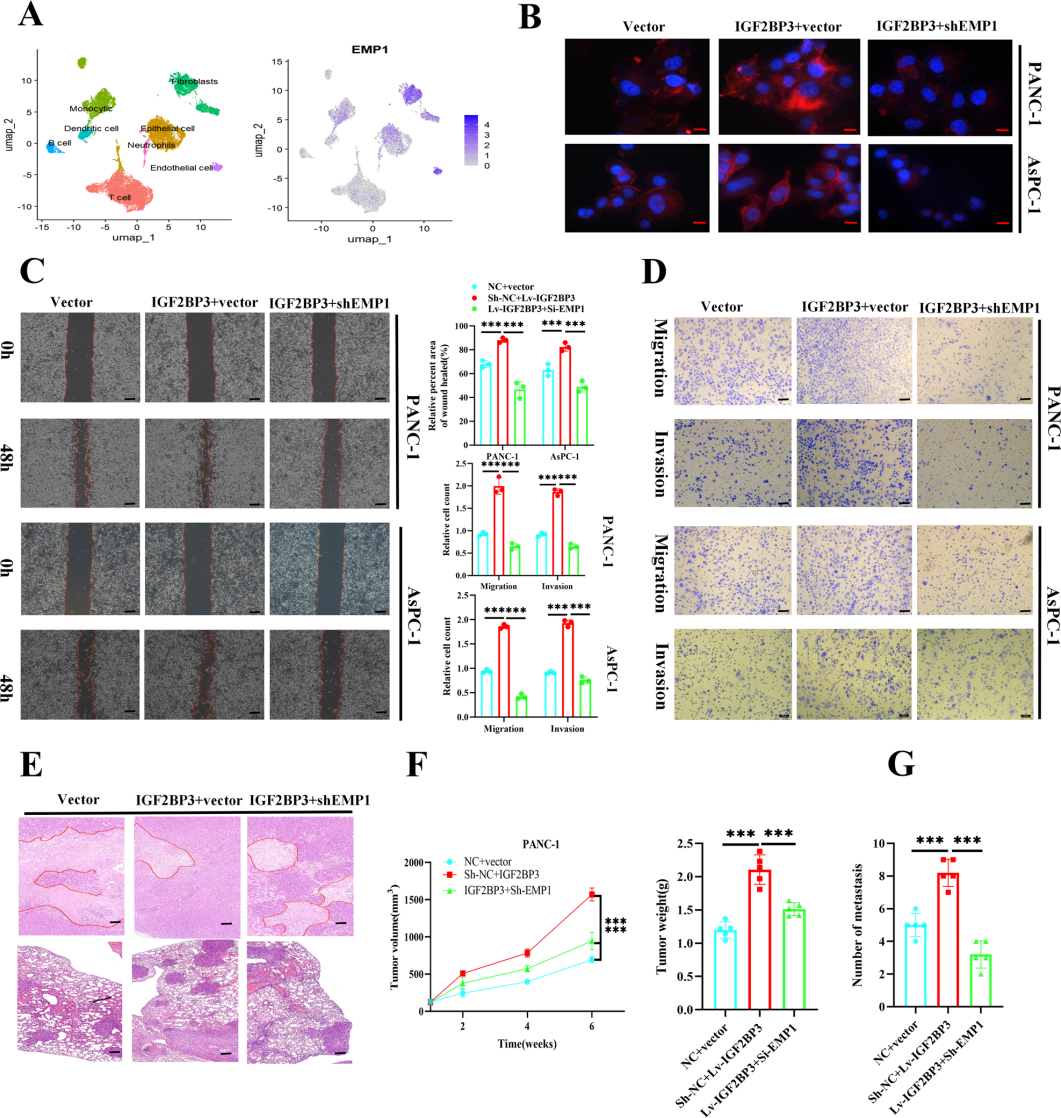
*IGF2BP3* combined with *EMP1* regulates the metastatic and invasive ability of pancreatic cancer cells in vitro and in vivo. Pancreatic cancer single-cell data reveal the subcellular division of *EMP1* in pancreatic cancer (**A**)**;** Assessment of the invasive ability of pancreatic cancer cells in Sh-control+vector, Sh-control+*IGF2BP3*, and *IGF2BP3* + Sh-*EMP1* groups: cytoskeletal staining (**B**) scratch assay (**C**) Transwell migration and invasion assay (**D**); Tumor H&E staining (**E**) proliferation curves (**F**) and weights (**F**) depict the morphology and tumor proliferation profile of subcutaneous tumor xenografts formed by the indicated cells. (1 × 10^6^), *n* = 5; statistical graph (**G**) depict lung metastases formed by tail vein injection of indicator cells, providing the number of lung surface nodules and metastatic lesions in mice lung tissue. *n* = 5. Scale: 100 μm. ***, *P* < 0.001.

We analyzed *EMP1* expression in single-cell populations of pancreatic cancer samples and found that *EMP1* was predominantly expressed in tumor cells (Fig. 5A). In pancreatic cancer cytoskeleton staining (Fig. 5B), scratch assay (Fig. 5C), and Transwell migration and invasion (Fig. 5D) assays, we found that the migratory and invasive abilities of pancreatic cancer cells, enhanced by *IGF2BP3* overexpression, were significantly reduced by *EMP1* depletion. As expected, subcutaneous xenograft tumors of pancreatic cancer cells overexpressing *IGF2BP3* revealed significantly enhanced tumor proliferation in vivo (Fig. 5E). However, this elevated proliferative ability of pancreatic cancer cells was attenuated by inhibiting *EMP1* expression (Fig. 5F, S2B). Depletion of *EMP1* also severely impaired the metastatic colonization of pancreatic cancer cells, overexpressing *IGF2BP3* in mice lung tissue when tumor cells were injected via the tail vein in nude mice (Fig. 5G, S2B).

In addition, we further explored the effects of knockdown of EMP1 and intervention of the m^6^A locus in the 3’UTR of EMP1 on the migratory and invasive ability of pancreatic cancer cells in in vivo and in vitro experiments. The results showed that knockdown of EMP1 inhibited the migratory and invasive ability of pancreatic cancer cells, and at the same time, the influence of knockdown EMP1 could be restored the migratory and invasive ability of pancreatic cancer cells through exogenous expression of the wild type of EMP1 rather than the mutant type of the m^6^A locus in EMP1 (Fig. S2C, D).

### *EMP1* promoted *TGF-β/Smads* signaling pathway activation by promoting the binding of *VASP* and *SMAD7*

**Fig. 6:**
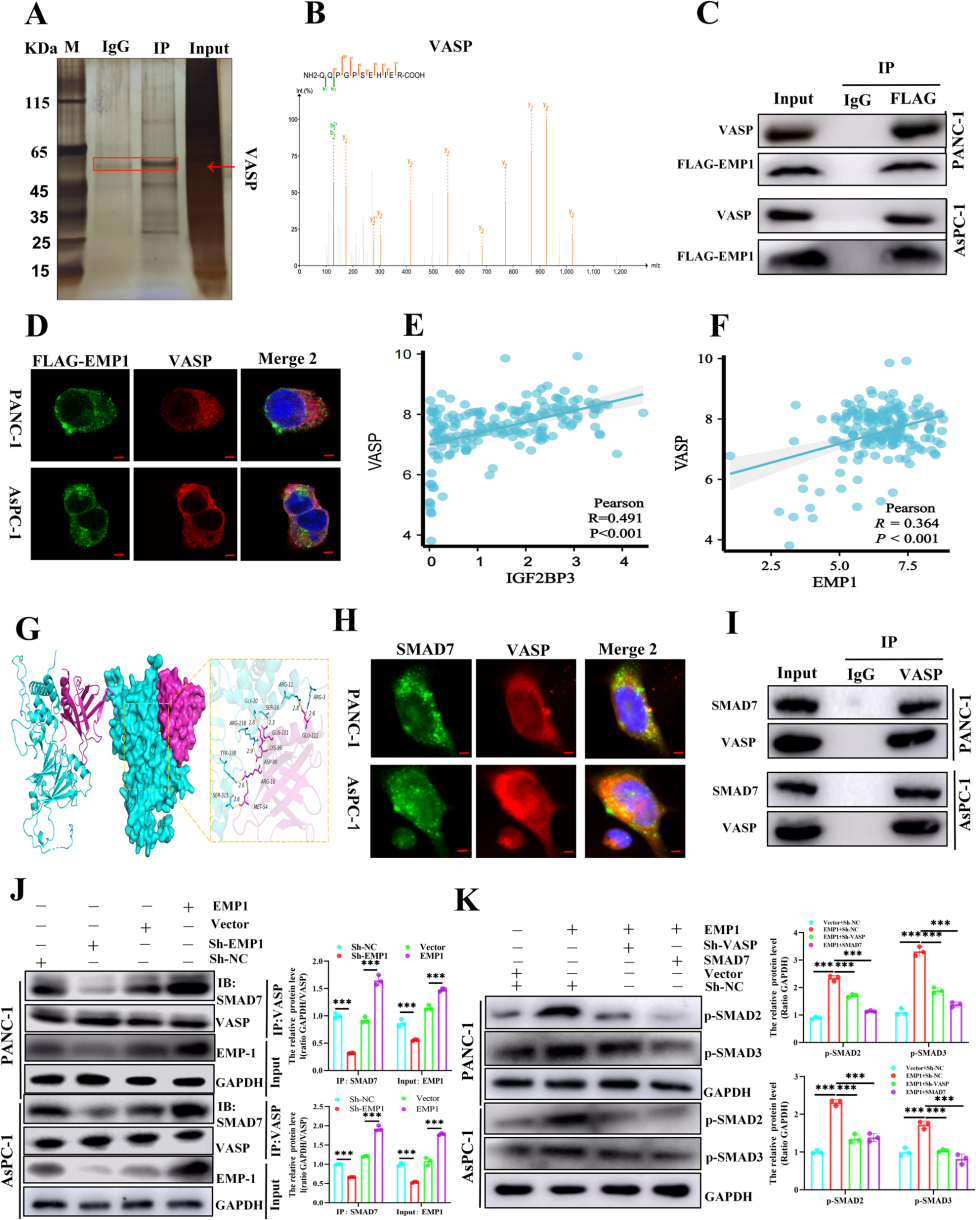
*EMP1* promoted *TGF-β/Smads* signaling pathway activation by promoting the binding of *VASP* and *SMAD7*. Immunoprecipitation assays obtain silver-stained bands of proteins binding to *EMP1-FLAG* (**A**); and LC-MS/MS analysis to demonstrate the binding sites of *EMP1* and *VASP* (**B**); In PANC-1 and AsPC-1, Co-IP experiments depict the interactions between *EMP1* and *VASP* (**C**); Immunofluorescence staining of PANC-1 and AsPC-1 demonstrating colocalization of *EMP1* and *VASP*, red (*VASP*), green (*EMP1*) and blue (*DAPI*) (**D**); Analysis of *IGF2BP3*, *EMP1*, with *VASP* correlation in the *TCGA-PAAD* dataset (**E**, **F**); Diagram of docking patterns of *VASP* and *SMAD7* proteins (**G**); Immunofluorescence staining of PANC-1 and AsPC-1 demonstrating colocalization of *VASP* and *SMAD7*, red (*VASP*), green (*SMAD7*) and blue (*DAPI*) (**H**); In PANC-1 and AsPC-1, Co-IP demonstrates the interactions of *VASP* and *SMAD7* (**I)** and *EMP1* expression affects this binding relation (**J);** Western blot detection of *EMP1-VASP-SMAD7* axis regulation of *TGF-β/Smads* signaling pathway in PANC-1 and AsPC-1 (**K)**. Scale: 20 μm. ***, *P* < 0.001.

Considering that *EMP1* is a quadruple transmembrane protein with intracellular and extracapsular binding ligands [27], we sought to determine which molecules bind to the intracellular region of *EMP1* to explore the downstream signaling of the *TGF-β/Smads* pathway mediated by *EMP1*. For this purpose, we screened an overexpression AsPC-1 of *FLAG-EMP1*, and the protein purified by the anti-*FLAG* antibody was detected by protein blotting and silver staining (Fig. 6A). Finally, *VASP* was identified by mass spectrometry as a protein that specifically binds to *EMP1* in pancreatic cancer cells (Fig. 6B). The interactions between *EMP1* and *VASP* were confirmed using co-immunoprecipitation (Co-IP) assays (Fig. 6C), and immunofluorescence staining confirmed the colocalization of *EMP1* and *VASP* in pancreatic cancer cells (Fig. 6D). Then, the correlation of *IGF2BP3*, *EMP1*, and *VASP* expression was analyzed in the *TCGA-PAAD* data cells, and results revealed that the expression of *EMP1* or *IGF2BP3* was positively correlated with *VASP* in pancreatic cancer (Fig. 6E, F). Previous studies have demonstrated that *VASP-like (EVL)* proteins can activate the *TGF-β/Smads* pathway by adhering to *SMAD7* [28]. We hypothesized that the structurally similar protein, *VASP*, plays a similar role in activating *TGF-β/Smads*. Therefore, we predicted a potential *VASP* interaction with the *SMAD7* protein using molecular docking experiments (Fig. 6G). Immunofluorescence staining confirmed the colocalization of *VASP* and *SMAD7* in pancreatic cancer cells (Fig. 6H). Besides, Co-IP analysis illustrated that in pancreatic cancer cells, *SMAD7* could bind to the *VASP* protein (Fig. 6I), and after knockdown of *EMP1* protein in pancreatic cancer cells, the ability of *VASP* protein binding to *SMAD7* was inhibited, and the reverse result was obtained by overexpression of *EMP1* (Fig. 6J). Subsequently, Western blot assay demonstrated that *VASP* knockdown reversed *TGF-β/Smads* signaling pathway activation resulting from *EMP1* overexpression. Consistently, when *SMAD7* overexpressed, the activation of the *TGF-β/Smads* pathway caused by *EMP1* was attenuated (Fig. 6K).

In conclusion, we found that the *EMP1*-promoted binding of *VASP* and *SMAD7* in pancreatic cancer promotes the activation of the *TGF-β/Smads* signaling pathway characterized by enhanced phosphorylation of *SMAD2/3* (Fig. 8). This provided a molecular theoretical basis for the design of drugs that inhibit the activation of the *TGF-β/Smads* pathway in tumor cells by modulating the function of the *EMP1-VASP* axis.

### Impact of *IGF2BP3* and *EMP1* on the tumor microenvironment of pancreatic cancer

**Fig. 7:**
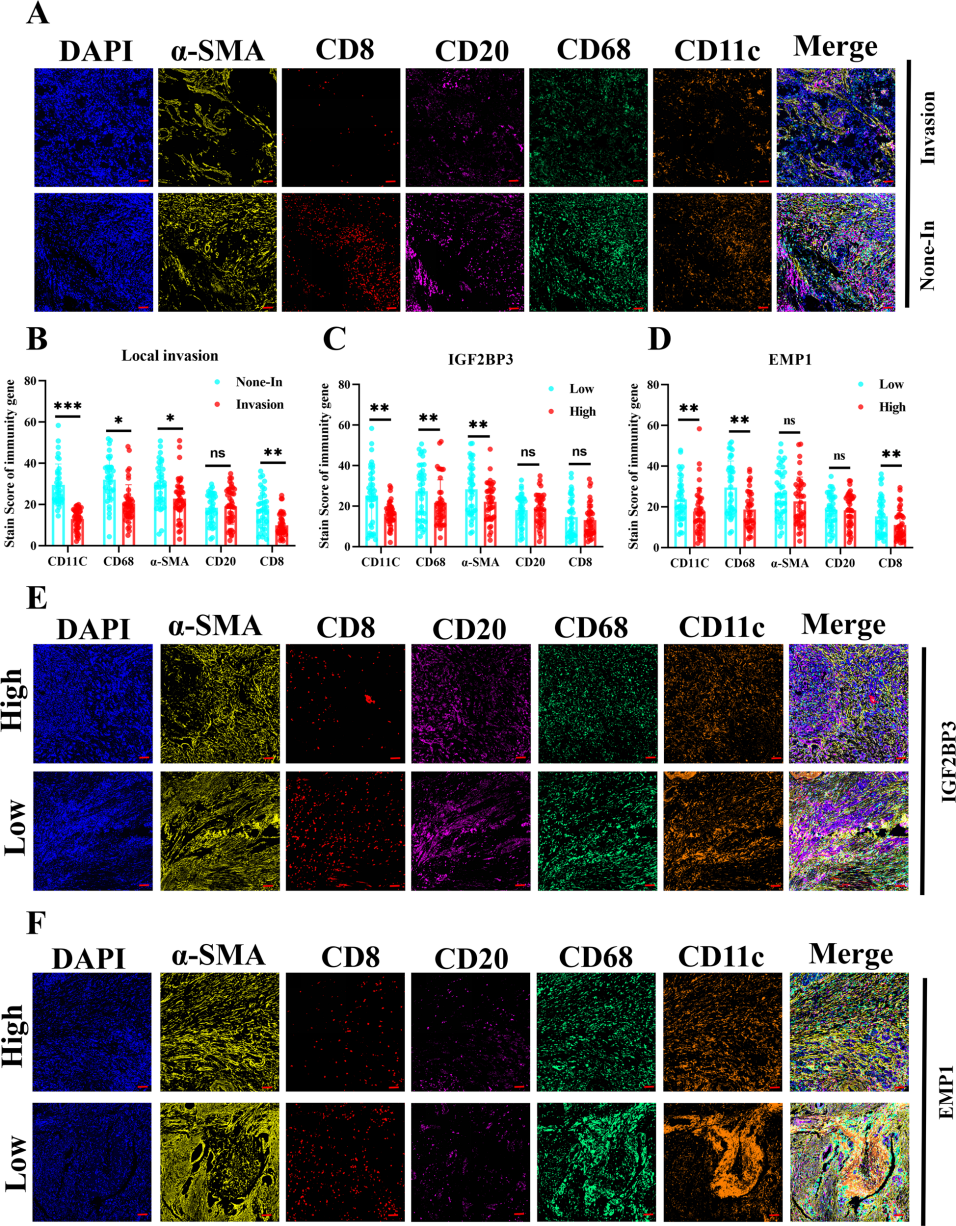
Effects of *IGF2BP3* and *EMP1* on the tumor microenvironment in pancreatic cancer. Multi-immunofluorescence staining reveals different immune microenvironment states. The proportion of *CD11c+* dendritic cells and *CD8* + T cells in pancreatic cancer tissues with localized invasion was significantly reduced (**A**, **B**). The levels of *IGF2BP3* (E) and *CD11c+* dendritic cells, *CD68+* macrophages, and α-SMA+ fibroblasts were closely correlated (**C, E**) and *EMP1* protein levels (**D**, **F**) were significantly correlated with infiltration of *CD11c+* dendritic cells, *CD68+* macrophages and *CD8* + *T* cells. Among them, *CD11c+* dendritic cells and *CD68+* macrophages showed significant changes upon local invasion, different expression states of *IGF2BP3* and *EMP1*. Scale: 100 μm. *, *P* < 0.05; **, *P* < 0.01; ***, *P* < 0.001.

Previous studies have expressed that m^6^A [15, 29] and *IGF2BP3* [30, 31] play important roles in regulating the tumor microenvironment and response to immune checkpoint drugs. In the current study, as a downstream target regulated by *METTL3-IGF2BP3*, *EMP1* is a multiple transmembrane protein that can communicate information with the tumor microenvironment [18, 32]. We further explored the role of *METTL3*, *IGF2BP3* and *EMP1* in the regulation of the tumor microenvironment in pancreatic cancer. We used serial-sectioned pancreatic cancer microarrays for multiple immunofluorescent labeling of major components of the tumor microenvironment, including *α-SMA, CD68, CD8, CD20*, and *CD11C* [33, 34]. Subsequently, infiltration-positive cells were analyzed according to the occurrence of local tissue invasion, *METTL3, IGF2BP3* and *EMP1* expression levels in pancreatic cancer (Figs. 7A, E, F, S2F). First, we observed that local invasion, *METTL3*, *IGF2BP3* and *EMP1* expression levels influence cell infiltration in the pancreatic cancer microenvironment(Fig. 7B–D; S2E). The infiltration of both *CD68*-labeled macrophages, which are the most predominant immune cells in the pancreatic cancer microenvironment [35, 36], and *CD11C*-labeled *DC*, which are essential for tumor cell antigen presentation [37], was significantly suppressed in the group with local invasion, *IGF2BP3*, or high *EMP1* expression (Fig. 7B–D). Moreover, *CD8* + *T* cells, which play an important role in antitumor immunity, were reduced in the infiltration of pancreatic cancer cells that underwent local invasion, high *METTL3* and high *EMP1* expression (Fig. 7B, D, S2E). The above results indicated that an immunosuppressive microenvironment was presented in pancreatic cancer with localized invasion and high expression of *IGF2BP3* and *EMP1*, which may be one of the reasons for the promotion of pancreatic cancer invasion.

## Discussion

**Fig. 8:**
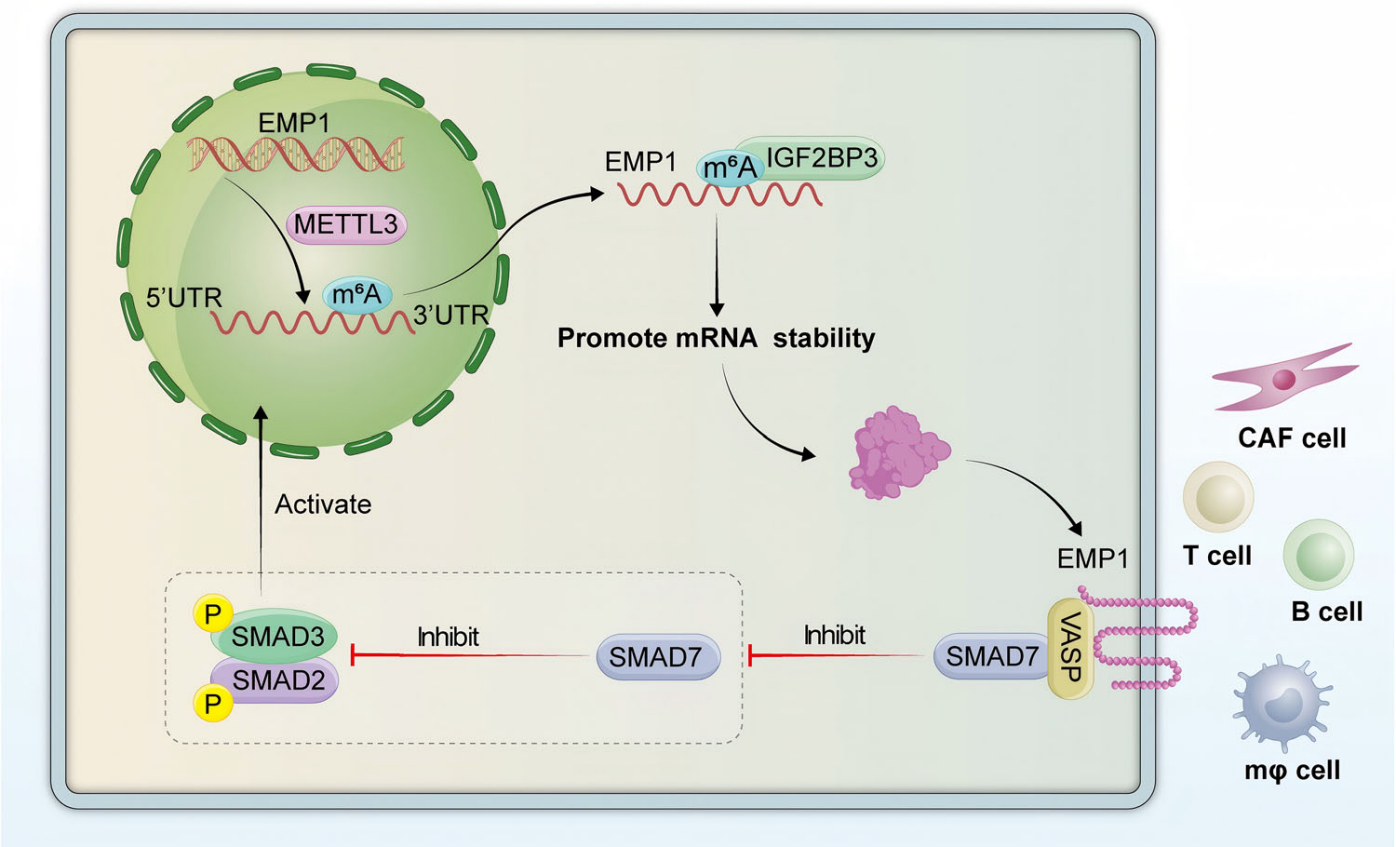
Schematic diagram revealing the regulatory mechanism of *IGF2BP3/EMP1/TGF-β.* *METTL3* mediates the modification of m^6^A on the *EMP1* RAN, and *IGF2BP3* promotes the stabilization of *EMP1 RNA* based on the recognition of the modification site of m^6^A on *EMP1*, and elevated *EMP1* protein levels, which increased *EMP1* and *VASP* binding. Meanwhile, the combination of *EMP1* and *VASP* enhanced the function of *VASP*, which is to impede the inhibition of the pathway of *TGF-β* signaling by *SMAD7*, ultimately leading to the sustained activation of *TGF-β* in pancreatic cancer. And the effect of *METTL3/IGF2BP3/EMP1* on the microenvironment of pancreatic cancer was also demonstrated, including: (*CD8* *+* T cell, *CD68+*Macrophage, *α-SMA+*fibroblast, *CD20* *+* B cell and *CD11c+*Dendritic cell).

*TGF-β* is vital for the development of tissue-specific diseases and progression to malignancy through the nuclear translocation of *SMAD* family members [38]. The importance of *TGF-β* signaling in PDAC is epitomized by over 50% frequency of genomic alterations in genes encoding proteins of the *TGF-β* pathway [39]. Besides, aggressive tumor-initiating cell population has been inhibited after *TGF-β* pathways blocking in PDAC [40]. Meanwhile, in clinical trials, tumor size and improved clinical response in relapsed metastatic pancreatic cancer were reduced when *PD-L1* antibody and *TGF-β* inhibitors were administered together [41]. However, how the *TGF-β/Smads* signaling pathway is regulated during tumor invasion and metastasis remains unclear. In the current study, we demonstrated that a specific metastatic promoter, *IGF2BP3*, confers a high invasive capacity to PDAC cells by inducing excessive activation of *TGF-β/Smads* signaling in the *EMP1/VASP* axis. Inhibition of the *IGF2BP3/EMP1/VASP* axis impaired the invasive and metastatic properties of PDAC cells (Fig. 8). These findings provide mechanistic insights into *TGF-β/Smads* signaling and tumor cell invasion during PDAC metastasis. The development of strategies to modulate the activation of the *TGF-β/Smads* signaling pathway by targeting the *IGF2BP3/EMP1/VASP* axis may hold promise to improve the current plight of pancreatic cancer.

Although m^6^A modification is crucial for tumor invasion and metastasis, how it shapes the invasive capacity of pancreatic cancer cells, specifically through the regulation of the *TGF-β/Smads* pathway, remains unidentified. m^6^A modification is dynamically and reversibly regulated by m^6^A “writers” and “erasers” in regulating the biological functions of tumors. Many studies have revealed that m^6^A modification plays a dual role in tumor development and progression. For example, the m^6^A writer *METTL3* exhibits both oncogenic and tumor-suppressive abilities in the same cancer type. Similarly, overexpression of the demethylase *FTO* promotes the progression of breast and colon cancers, whereas *FTO* deletion induces EMT and promotes breast cancer metastasis. Based on these results, we recognized that the m^6^A “writers,” represented by *METTL3* or *METTL14*, provide the total basic regulatory pattern at the RNA level during tumorigenesis and that the fate of m^6^A-modified RNAs, such as RNA stability or translational efficiency, which is usually regulated by m^6^A “readers,” may play a significant role in determining the expression level of m^6^A-modified RNA. We observed that elevated levels of *METTL3* in invasive pancreatic cancer provided a general m^6^A modification motif for RNA. Another key “reader,” *IGF2BP3*, which is the most significantly upregulated of all m^6^A-related genes in aggressive PDAC tumors, activates the *TGF-β/Smads* pathway in an m^6^A-dependent manner to make PDAC cells highly invasive and metastatic. *IGF2BP3* recognizes m^6^A-sites of *EMP1* mRNA, promotes its stability, and upregulates *EMP1* protein levels in an m^6^A-dependent manner. Elevated *EMP1* inhibits the interference of *SMAD7* with *SMAD2/3* activity by promoting the binding of *VASP* to *SMAD7*, ultimately activating the *TGF-β/Smads* signaling pathway-induced cancer cell invasiveness (Fig. 8). Taken together, our current study identified a critical role for *IGF2BP3* and m^6^A modification in tumor invasion and subsequent metastasis. Consequently, targeting m^6^A modification by inhibiting *IGF2BP3* may be a potentially valuable therapeutic strategy for the treatment of early invasion in PDAC.

In the tumor microenvironment, the successful formation of an invasive niche depends on the tumor cell’s invasive and migratory abilities and the formation of a dysfunctional immunosuppressive microenvironment, which is formed by the interactions of the tumor cells and tumor-associated fibroblasts [42], macrophages [43], T-cells [44], neutrophils [45], and NK-cells [46]. Previous studies have demonstrated that m^6^A modification plays an important role in regulating intercellular communication in the tumor microenvironment, for example, orchestrates [47], colon [48], and non-small cell lung cancer [49]. Moreover, the exchange of information and signaling between cells in the microenvironment mainly relies on chemical signaling molecules, which are the most commonly used communication methods, followed by neighboring cell surface molecular adhesion and cell adhesion to the extracellular matrix [50]. *EMP1*, a multiple transmembrane protein, has an information reception/transduction ligand in the extramembrane and intramembrane [19, 51]. These interesting clues prompted us to further analyze the correlation between *IGF2BP3-EMP1* and the tumor microenvironment in clinical samples of pancreatic cancer. We found that local tissue invasion and the level of *IGF2BP3* and *EMP1* affected the immune cell infiltration in pancreatic cancer by multiple immunofluorescence staining. Macrophages, which account for the largest proportion of pancreatic cancer tissues and DC cells responsible for antigen presentation, were consistently inhibited from infiltrating in samples with local tissue invasion and high levels of *IGF2BP3* and *EMP1*. Besides, our study found that CD8 + T cells were suppressed in local tissue infiltration and *EMP1* high-expression samples, demonstrating that an immunosuppressive microenvironment exists in local invasion, *IGF2BP3*, and *EMP1* high-expression pancreatic cancer tissues, which may explain the deterioration of pancreatic cancer invasion by *IGF2BP3-EMP1* (Fig. 8).

In conclusion, as shown in the Fig. 8, this study reveals a critical role of the m^6^A reader protein *IGF2BP3* in the local invasion of PDAC. By recognizing and stabilizing m^6^A-modified *EMP1* mRNAs, *IGF2BP3* promotes *EMP1* protein expression, which in turn activates the *TGF-β* signaling pathway, thereby enhancing the invasive properties of PDAC cells. This m^6^A-dependent mechanism not only contributes to the poor prognosis of patients but also shapes the immune microenvironment within the tumor. These findings underscore the potential of targeting the *IGF2BP3-EMP1* axis as a therapeutic strategy to impede local invasion and metastasis in PDAC.

## Supplementary information

Supplementary legends

Figure S1

Figure S2

Figure S3

Table S1

## Data Availability

All datasets generated and analysed during this study are included in this published article and its Supplementary Information files. Additional data are available from the corresponding author on reasonable request.
