# Supplementary material for: IGF2BP3 regulates EMP1 stability in an m6A-dependent manner and activates the TGF-β pathway to promote pancreatic cancer invasion
Source: Cell Death Dis. 2025 Nov 24;16(1):858. doi: 10.1038/s41419-025-08155-1 (PMC12645055; doi:10.1038/s41419-025-08155-1)
Supplement: Supplementary file 1 — Supplementary legends [file 41419_2025_8155_MOESM1_ESM.docx]

**Figure S1. IGF2BP3 regulates m6A modification and EMP1 expression in pancreatic cancer**

Western blot detection of METTL3 expression in pancreatic cancer tissues (A); Cohort1 analysis of the effect of METTL3 expression on overall survival time in pancreatic cancer(B); Volcano plot demonstrating Sh-NC and *Sh-IGF2BP3* group differential genes in PANC-1 (C); Cell population marker of Single-cell dataset of pancreatic cancer (D)；Statistical plots of Western blot detection of EMP1 protein expression in the Sh-NC, Sh-IGF2BP3, Vector and IGF2BP3 groups, and EMP1 protein expression in the Si-NC+Vector, METTL3+Si-NC, METTL3+Si1-IGF2BP3 and METTL3+Si2-IGF2BP3 groups in PANC-1 and AsPC-1(E); Representative diagram of subcutaneous xenograft tumor and lung metastasis model in nude mice in *Sh-NC, Sh-IGF2BP3*, Vector and *IGF2BP3* groups (F);The effects of METTL3-IGF2BP3 on the migration and invasion capabilities of pancreatic cancer cells were assessed through cytoskeletal staining, scratch healing assay, and Transwell assay. In vivo experiments were used to evaluate the effects of METTL3 expression levels on the proliferation capabilities of pancreatic cancer cells. (G); In vivo experiments to assess the effect of METTL3-IGF2BP3 on pancreatic cancer proliferation(H); analysis of m^6^A modification sites based on SRAMP (an online m^6^A site predictor) prediction of the m6A site of *EMP1* (I); RNA pull-down assay detects binding of the m^6^A site in EMP1 to the EMP1 mRNA GF2BP3 protein (J); Statistical plot of Western blot detection for *IGF2BP3* in HPDE and pancreatic cancer cell lines (K)；Statistical plots of Western blot detection of *EMP1* protein expression in the Sh-NC, *Sh-IGF2BP3*, Vector and *IGF2BP3* groups in PANC-1 and AsPC-1 (L); Statistical plots of Western blot detection of *EMP1* protein expression in the Si-NC+Vector, *METTL3+Si-NC*, *METTL3+Si1-IGF2BP3* and *METTL3+Si2-IGF2BP3* groups in PANC-1 and AsPC-1 (M). Scale: 100 μm. *, *P* < 0.05; **, *P* < 0.01; ***, *P* < 0.001.

**Figure S2. Expression patterns of METTL3, IGF2BP3, EMP1 and VASP in pancreatic cancer and their role in the tumour microenvironment**

Expression of *METTL3*, *IGF2BP3*, *EMP1* and *VASP* in single-cell samples of pancreatic cancer(A)；Typical diagram of subcutaneous xenograft tumor and lung metastasis model in nude mice in *Vector+Sh-NC*, *IGF2BP3+Sh-NC* and *IGF2BP3+Sh-EMP1* groups(B)；Cytoskeletal staining, scratch healing assay, and Transwell assay to evaluate the effect of EMP1 and m^6^A modification on the migration and invasive ability of pancreatic cancer cells(C); In vivo experiments to assess the effect of EMP1 and m6A modification on pancreatic cancer proliferation(D); Multi-immunofluorescence staining reveals the levels of *METTL3* (F) on the cell infiltration status of the pancreatic cancer microenvironment, and the statistic graphs (E). Scale: 100 μm. *, *P* < 0.05; **, *P* < 0.01; ***, *P* < 0.001.
