# Supplementary material for: IGF2BP3 regulates EMP1 stability in an m6A-dependent manner and activates the TGF-β pathway to promote pancreatic cancer invasion
Source: Cell Death Dis. 2025 Nov 24;16(1):858. doi: 10.1038/s41419-025-08155-1 (PMC12645055; doi:10.1038/s41419-025-08155-1)

Figure1

D

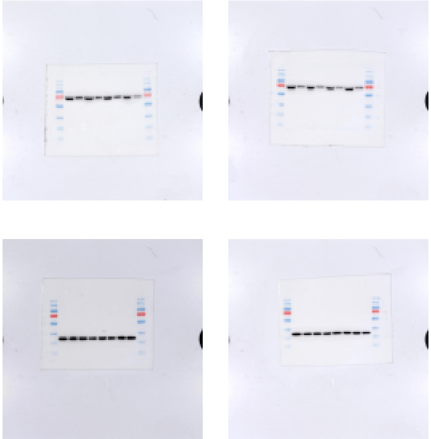

I

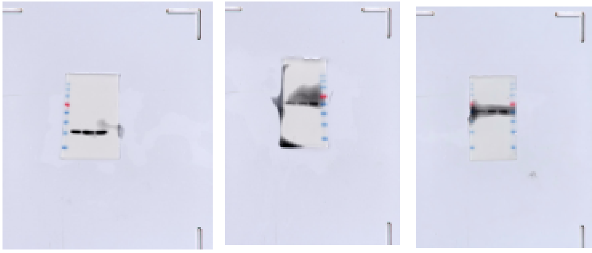

J

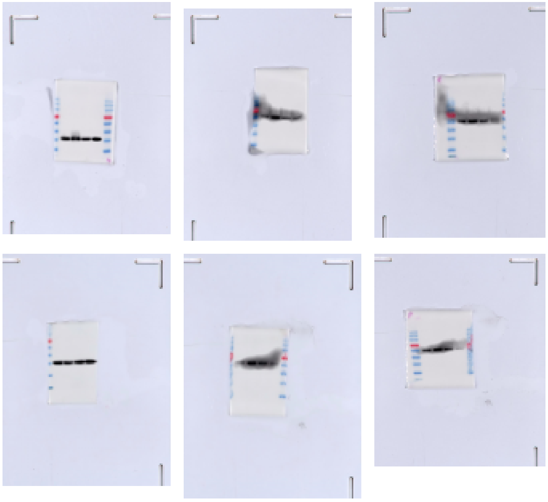

Figure 2B

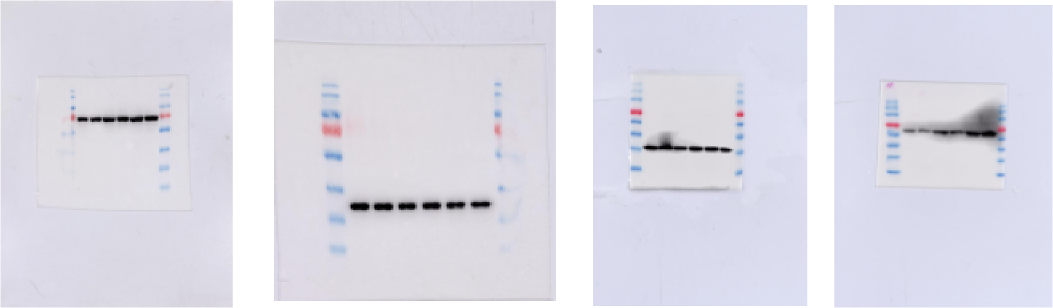

Figure 3C

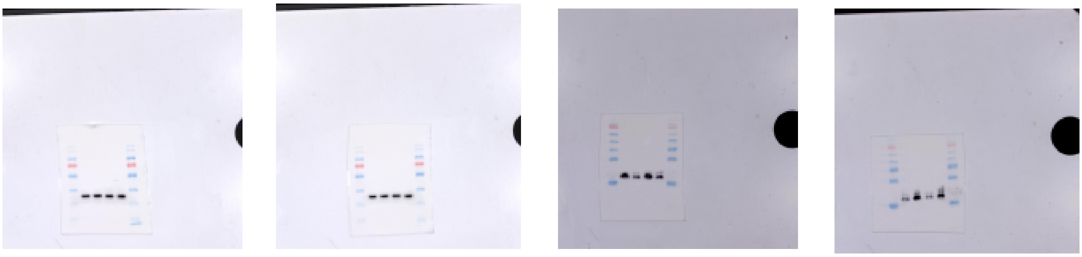

Figure 3H

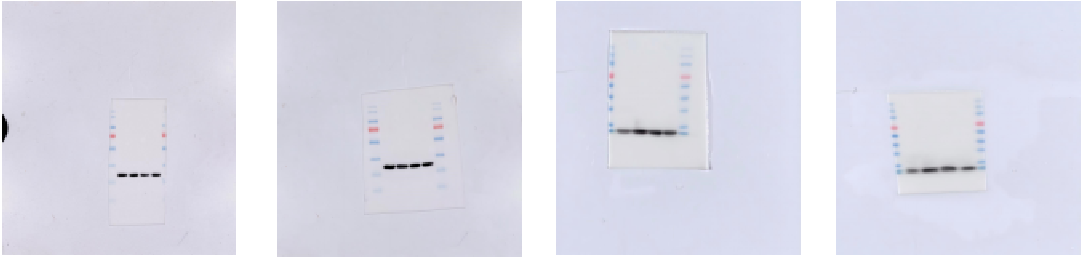

Figure 3I

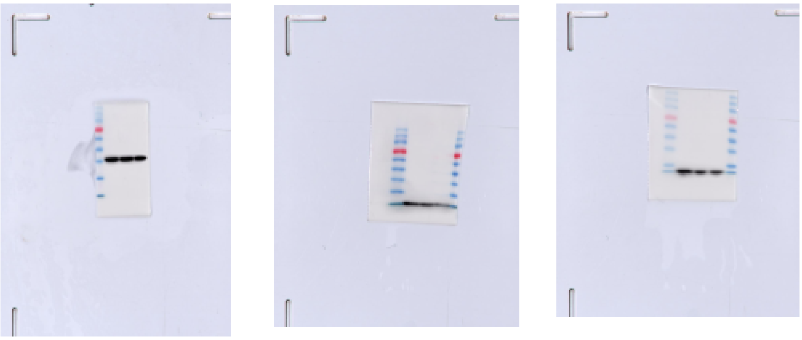

Figure4E

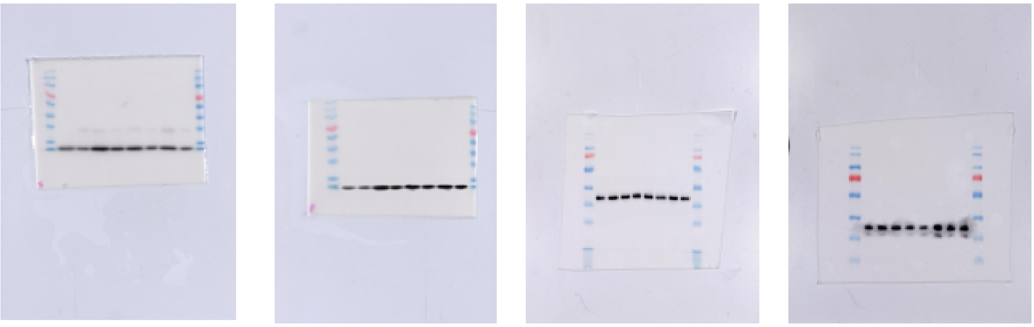

Figure4H

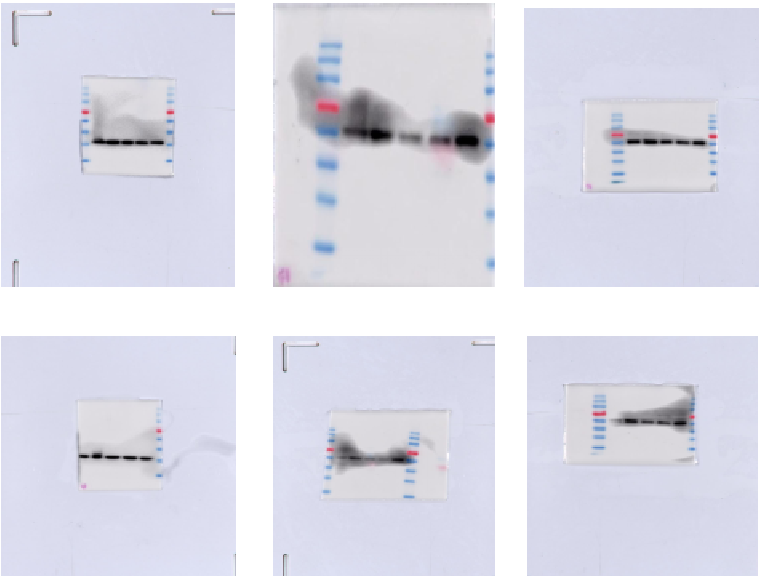

Figure6

A

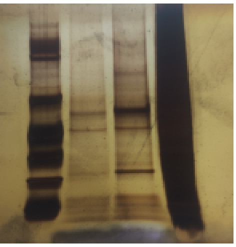

C

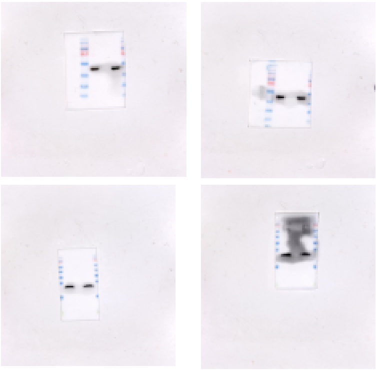

I

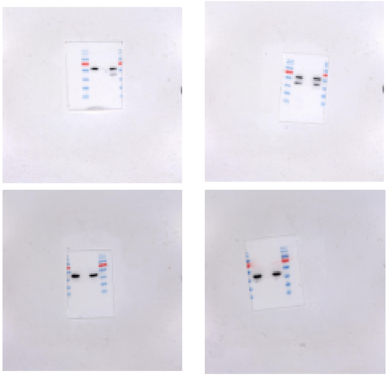

Figure6 J

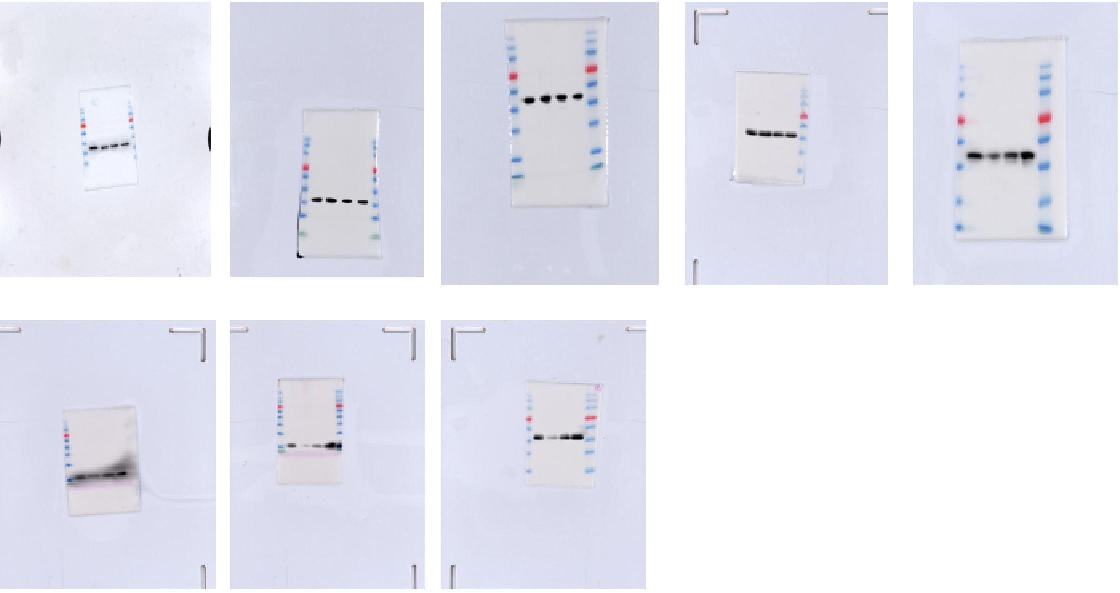

Figure6 K

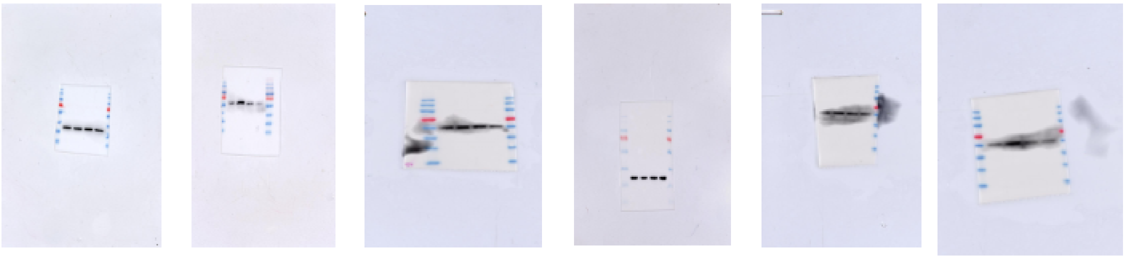

Figure S1

A

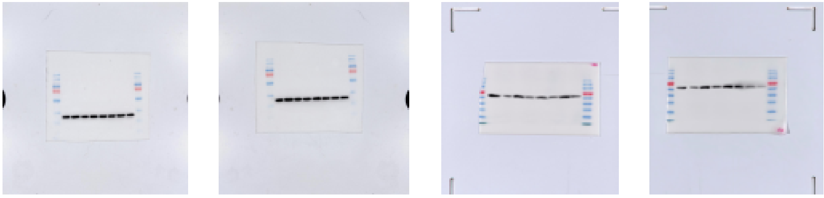

J

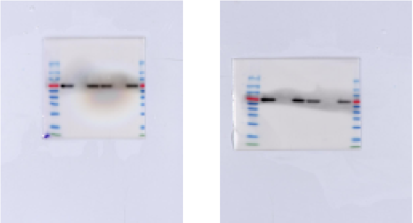

Supplement: Supplementary file 4 — Figure S3 [file 41419_2025_8155_MOESM4_ESM.pdf]
